# Anticancer effects and mechanisms of *Pulsatilla chinensis*, *Bupleurum chinense* and *Polyporus umbellatus* on human lung carcinoma and hepatoma cells

**DOI:** 10.1016/j.csbj.2025.07.023

**Published:** 2025-07-13

**Authors:** Mengzhen Li, Woonghee Kim, Han Jin, Hong Yang, Xiangtai Kong, Xiya Song, Hasan Turkez, Yuefeng Bi, Chengxue Pan, Ling Fu, Hongmin Liu, Mathias Uhlen, Cheng Zhang, Adil Mardinoglu

**Affiliations:** a Science for Life Laboratory, KTH – Royal Institute of Technology, Stockholm SE-17165, Sweden; b Central Laboratory, Tianjin Medical University General Hospital, Tianjin, China; c Department of Medical Biology, Faculty of Medicine, Atatürk University, Erzurum 25240, Turkiye; d School of Pharmaceutical Sciences, Zhengzhou University, Zhengzhou, Henan Province, China; e Key Laboratory of Advanced Drug Preparation Technologies, Ministry of Education, School of Pharmaceutical Sciences, Zhengzhou University, Zhengzhou, Henan Province, China; f Centre for Host-Microbiome Interactions, Faculty of Dentistry, Oral & Craniofacial Sciences, King’s College London, London SE1 9RT, UK

**Keywords:** Traditional Chinese medicine, Herbal extract, Liver cancer, Lung cancer, Cancer therapeutics, RNA sequencing, Bioinformatic analysis

## Abstract

Herbs are extensively utilized in Traditional Chinese Medicine (TCM) for lung and liver cancer treatment, but the mechanisms behind these herbs remain largely unknown. Here, high-throughput transcriptomic analysis technology was used to uncover molecular mechanisms of herbal treatment. Furthermore, we developed a compound recognition approach utilizing the LINCS L1000 database to identify potential treatment targets. Our results showed that among 14 herbs tested, *Pulsatilla chinensis* exhibited the strongest anticancer effects in A549 and Huh7 cells, followed by *Bupleurum chinense*, and *Polyporus umbellatus*. *P. chinensis* exerts its anticancer properties by downregulating cell cycle-related transcription factors, including E2F1 and TFDP1. Notably, the mechanisms of *P. chinensis* treatment differed between the two cell lines. In A549 cells, which possess wild-type p53, *P. chinensis* induced apoptosis through the regulation of the p53 pathway. In contrast, in Huh7 cells, which harbor mutant p53, the effect was mediated via the TNF-α/NF-κB signaling pathway. We also identified two drugs, AMG232 and Nutlin-3, that exhibited treatment effects similar to *P. chinensis* in A549 cells. Both drugs function as inhibitors of the MDM2-p53 interaction. Western blot analysis confirmed the alteration of the relevant proteins, aligning with our computational predictions. Furthermore, 23-hydroxybetulinic acid, a key active compound of *P. chinensis*, demonstrated the ability to inhibit the p53-MDM2 interaction by binding to the same pocket on the MDM2 protein.

## Graphical Abstract

**Figure:**
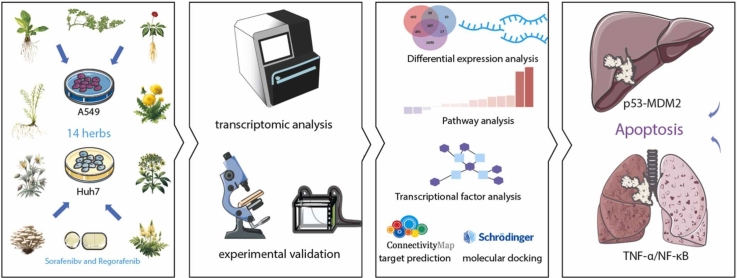

## 1 Introduction

Cancer has a significant impact on global health [1]. Liver and lung cancers are among the most common cancer types and remain leading causes of cancer-related deaths worldwide [2], [3]. Effective treatment remains a significant challenge [4]. According to 2023 data from the American Cancer Society, the 5-year relative survival rate is 23 % for lung cancer and 21 % for liver cancer [5]. Current therapeutic options for lung and liver cancers mainly rely on surgical removal, chemotherapy, radiation therapy, and immunotherapy [6], [7]. However, the efficacy of these treatments is limited, and there are even fewer options for advanced cancers. Additionally, patients receiving available therapies often experience a range of side effects shortly after the initial dose. For instance, immunotherapy may lead to autoimmunity and nonspecific inflammation [8]. Chemotherapy could induce multidrug resistance (MDR) [9]. Consequently, the development of new drugs and treatment strategies is essential for advancing cancer treatment.

Natural compounds play an important role in cancer therapy [10]. Drug discoveries from medicinal plants continue to provide new and effective therapeutic options against various pharmacological targets, including those for cancers [11]. Plant-derived drugs are widely used in traditional Chinese medicine (TCM), and long-term practice has shown that some of them possess anticancer properties. However, due to their complex nature and intricate composition, it remains challenging to systematically understand the mechanisms of action and identify therapeutic targets of herbs in cancer treatment. Advancements in high-throughput sequencing and computational biology have made it feasible to apply transcriptomic and bioinformatics analyses across a wide range of fields. These approaches are effective for studying the alteration of gene-expression profiles induced by herbal treatment, which could reveal the overall biological effects of herbs. This aids in a more thorough exploration of the underlying mechanisms of herbs in cancer therapy [12].

In this study, we aimed to identify the herbs that have potential anticancer effects on lung and liver cancer and reveal their underlying molecular mechanisms based on global transcriptomic analysis. The selected cell models for our study were human A549 lung carcinoma, the most representative human non-small cell lung cancer cell line [13], [14], and Huh7 hepatocellular carcinoma, which is also a commonly used liver cancer cell line, showing high concordance of gene expression with human hepatoma [15]. We then evaluated the efficacy of 14 commonly used anticancer herbs on the cell viability of A549 cells and Huh7 cells. These 14 herbs are categorised under 9 different plant families and fall within the six main categories of therapeutic effects according to TCM theory for cancer treatment (Table 1). Based on cell viability results, the most effective herbs were selected for further analysis. RNA was extracted from A549 and Huh7 cells following treatment with these herbs and subjected to RNA sequencing. Bioinformatics analyses, including differential gene expression, functional enrichment, and transcription factors (TFs) prediction, were conducted to uncover the mechanisms underlying the therapeutic effects of the herbal treatments. In addition, by integrating computational analyses with the LINCS perturbation-based gene expression database, we identified pharmaceutical compounds that share similar mechanisms of action with the studied herbs based on transcriptomic profiles. Finally, experimental validation was conducted to confirm the pathways identified through transcriptomic analysis. In summary, this comprehensive study combined computational and experimental approaches to elucidate the molecular mechanisms underlying the effectiveness of selected herbs in the treatment of lung and liver cancers.

**Table 1:** 14 Herbs included in this study.

TCM categories	Plant families	Official name	Abbreviation	Chinese name
heat-clearing	Ranunculaceae	*Pulsatilla chinensis* (Bunge) Regel	*P.chinensis*	BaiTouWeng
	Rubiaceae	*Scleromitrion diffusum* (Willd.) R.J.Wang	*S.diffusum*	BaiHuaSheSheCao
	Brassicaceae	*Isatis indigotica* Fort.	*I.indigotica*	DaQingYe
	Asteraceae	*Taraxacum mongolicum* Hand.-Mazz.	*T.mongolicum*	PuGongYing
	Asteraceae	*Artemisia annua* L.	*A.annua*	QingHao
immunity enhancement	Campanulaceae	*Codonopsis pilosula* (Franch.) Nannf.	*C.pilosula*	DangShen
	Fabaceae	*Glycyrrhiza uralensis* Fisch.	*G.uralensis*	GanCao
	Fabaceae	*Astragalus membranaceus* Fisch	*A.membranaceus*	HuangQi
	Araliaceae	*Panax ginseng* C.A.Mey.	*P.ginseng*	RenShen
diuretic properties	Polyporaceae	*Poria cocos* (Schw.) Wolf	*P.cocos*	FuLing
	Polyporaceae	*Polyporus umbellatus* (Pers.) Fries	*P.umbellatus*	ZhuLing
liver soothing	Apiaceae	*Bupleurum chinense* DC.	*B.chinense*	ChaiHu
Yang-tonifying	Ranunculaceae	*Aconitum carmichaelii* Debeaux	*A.carmichaelii*	HeiShunPian
blood stasis removal	Asteraceae	*Carthamus tinctorius* L	*C.tinctorius*	HongHua

## 2 Materials and methods

### 2.1 Materials and reagents

A549 and Huh7 cell lines were obtained from the Cell Profiling Unit at the Science for Life Laboratory. Cells were maintained with DMEM High glucose (D0822, Sigma-Aldrich) supplemented with 10 % fetal bovine serum (F7524, Sigma-Aldrich), and 1 % P/S (P4333, Sigma-Aldrich). HepG2 and H1299 cells was obtained from Cytion and HepG2. Cells were cultured with RPMI-1640 (R2405, Sigma-Aldrich) supplemented with 10 %FBS and 1 % P/S.

Fourteen herbs were used in this study. All of them were purchased from Henan Yunfeng Pharmaceutical Co., Ltd. in Yuzhou City, China. Voucher specimens of *S.diffusum* (2019–29), *P.chinensis* (2019–18), *B.chinense* (2019–13), *C.pilosula* (2019–15), *I.indigotica* (2019–17), *P.cocos* (2019–20), *G.uralensis* (2019–28), *C.tinctorius* (2019–16), *A.carmichaelii* (2019–23), *A.membranaceus* (2019–27), *T.mongolicum* (2019–24), *A.annua* (2019–25), *P.ginseng* (2019–26), *P.umbellatus* (2019–19) were deposited at the Zhengzhou University School of Pharmacy Specimen Museum. These plant names have been checked with http://mpns.kew.org.

To obtain the herbal extracts, herbs were first pulverized and filtered through a screen with a pore size of 6.6 mm and a mesh count of 3. Then, it was immersed in 75 % ethanol for 30 min at 40°C. After that, the ultrasonic extraction was performed twice, each lasting one hour, at 40°C for flowers and whole plane and 50°C for rhizomes. Then, the mixture underwent vacuum concentration at 50°C. Density after concentration is 1.0–1.1 g/ml. Thin-layer chromatography identification was utilized to check whether the active compounds were thoroughly extracted. The concentrated extract was frozen at −40°C in a freezer for 24 h and then subjected to freeze-drying under vacuum for 48 h. The resultant freeze-dried powder was then reconstituted in DMSO to a concentration of 10 mg/ml for subsequent experiments.

### 2.2 Cell viability assay

For the cell viability assay, cells were seeded at 5000 cells per well into 96-well plates as triplicated. For anticancer toxicity screening, we treated 10 µg/ml herbal extracts to cells for 48 hr. Additionally, IC_50_ value measurements were performed using concentrations of 30, 10, 3, 1, 0.3, 0.1, and 0.03 µg/ml, respectively. Sorafenib and Regorafenib were used as µM and same digit, respectively. Cell viability was measured by MTT assay (M6494, ThermoFisher) by following the manufacturer’s instructions. Optical density (OD) was enumerated with a microplate reader (Hidex Sense Meta Plus).

### 2.3 Western blot analysis

Cells were seeded into a 6-well plate at 200,000 cells per well. After 10 µg/ml herbal extract treatment, whole cell lysate was prepared with CelLytic M (C2978, Sigma-Aldrich) buffer, and samples were prepared with 2x Laemmli Sample Buffer (1610737, Biorad) at 20 µg protein lysate. SDS PAGE was performed with Mini-PROTEAN® TGX™ Precast Gels (Bio-Rad) and transferred using Trans-Blot® Turbo™ Transfer System (Bio-Rad). PARP (9542S, Cell signaling), Active Casapse3 (ab214430, Abcam), Active caspase8 (D374, Cell signaling), FADD (ab124812, Abcam), MDM2 (33–7100, Invitrogen), GAPDH (ab8245, Abcam), p53 (ab32389, Abcam), c-Myc (ab32072, Abcam), Histon H3 (ab1791, Abcam), NF-κB p65 (ab16502, Abcam), NF-κB P50 (AB32360, Abcam), DP1 (MA5–35676, Invitrogen) and α-tubulin (ab7291, Abcam) were blotted as a primary antibody overnight. Secondary antibodies, Goat Anti-Rabbit HRP (ab205718) and goat anti-mouse IgG-HRP (sc2005, Santa Cruz Biotechnology, Inc.) were blotted for one hour. The protein band was detected with ImageQuantTMLAS 500 (29–0050–63, GE).

### 2.4 RNA extraction and sample preparation for RNA sequencing

RNA extraction was performed using the RNeasy Plus Mini Kit (74134, QIAGEN). The integrity of the extracted RNA was assessed by measuring the RNA Integrity Number (RIN) using the RNA 6000 Nano kit (5067–1511, Agilent) on the 2100 Bioanalyzer Instrument (G2939BA, Agilent), which utilizes electrophoresis for this measurement. Samples with RIN scores above 8 were selected and sent to SZA Omics in Istanbul for RNA sequencing analysis.

### 2.5 Library preparation and RNA-sequencing

The quality and quantity of the samples were assessed spectrophotometrically with NanoDrop (Thermo Fisher, USA). For the RNA samples, the ratio of absorbance at 260 and 280 nm (A260/280) of ∼2, A260/230 of 2.0 – 2.2, and RIN > 8.0 were accepted as acceptable quality.

Stranded Total RNA Prep, Ligation with Ribo-Zero Plus kit is used for the construction of NGS libraries from the samples with acceptable quality parameters. RNA samples were sequenced 2 × 100 paired-end with a total of 25 M fragment reads. Pair-end sequencing was performed by NovaSeq6000 (NovaSeq Control Software 1.6.0/ RNA v3.4.4).

Raw sequencing data (.bcl) was demultiplexed and converted to FASTQ with DRAGEN Software, v3.9.5. The data was converted to fastq format using Illumina 1.8 quality scores.

### 2.6 RNA-seq data preprocessing

The fastq files were first explored by FastQC (Babraham Institute, Cambridge, UK) for quality control. Gene expression count data was quantified using the standard protocol of Kallisto [16]. The reference cDNA for alignment and quantification was GRCh38.p13 (v108) for Homo sapiens, which was retrieved from the Ensembl website. After filtering only protein-coding genes and genes with an average count greater than 5, the Kallisto data was used for the downstream analysis. There were 13776 and 14107 genes retained in the A549 and Huh7 cell groups, respectively.

### 2.7 Differential Expression Analysis

We used the DESeq2 R package (v1.36.0) [17] to identify the differentially expressed genes (DEGs). Significance for up-regulated genes was determined with an adjusted P-value < 0.05 and a log_2_ fold change > 0, and vice versa (log_2_ fold change < 0) for down-regulated genes. The Benjamini-Hochberg (BH) correction was used for multiple testing corrections.

### 2.8 Principal Component Analysis

Based on gene expression profiles after variance stabilizing transformation, Principal Component Analysis (PCA) was used to explore the sample distribution using the R package of pcaMethods (v1.92.0) [18].

### 2.9 Gene set functional analysis

We performed gene set enrichment analysis (GSEA) [19] to assess the associated biological pathways in the A549 and Huh7 cells after *P. chinensis, B. chinense* and *P. umbellatus* treatments. For this, genes were sorted by log_2_ fold change in descending order, and hallmark gene sets from the Molecular Signatures Database (MSigDB) [20] were tested for their significance.

In addition, gene set overrepresentation analysis (GSOA) was applied to determine whether known biological functions or processes were overrepresented in the DEGs induced by herbal treatments. To understand the biological mechanism of different herbal treatments, we extracted the up-regulated and down-regulated genes from *P. chinensis, B. chinense* and *P. umbellatu* treatment groups in both A549 and Huh7 cells. We used these extracted lists of genes as interested genes and all detected genes as the background to explore whether genes of interest were significantly associated with specific Gene Ontology (GO) biological process terms. Further, to explore the biological functions of genes commonly modulated by all three herbal treatments, we identified the common genes that were either up-regulated or down-regulated across treatment with *P. chinensis, B. chinense* and *P. umbellatus* in both A549 and Huh7 cells. These common genes were then analyzed via GSOA to uncover any significant association with biological functions.

The R package clusterProfiler (v4.4.4) [21], [22] was used for both GSOA and GSEA.

### 2.10 DEG similarity comparison

We used the Jaccard index to assess the similarity among the DEGs across various treatment groups, which is defined as the size of the intersection divided by the size of the union of two gene sets. We also applied the R package GOSemSim (v2.22.0) [23] to perform the semantic comparisons of significant GO biological processes associated with different DEG sets, which could quantify the similarity of two sets of enriched GO terms.

### 2.11 Transcription factors prediction

We used ChIP-X Enrichment Analysis 3 (ChEA3), a web-based tool for transcription factors enrichment analysis (TFEA) [24]. ChEA3 libraries contain a collection of TF-target gene set libraries from multiple sources, including ChIP-seq experiments, TF–gene co-expression from RNA-seq studies, and TF–gene co-occurrence computed from crowd-submitted gene lists. By comparing a set of DEGs with ChEA3 libraries using Fisher's exact test, this tool can aid in predicting the transcriptional factors (TFs) responsible for the query DEGs. In our study, the DEGs of different treatment groups were submitted to ChEA3 as input. The top ten TFs were selected by mean rank across all libraries. We selected representative TFs along with their regulated genes to construct the gene regulatory network. To understand the biological functions of the selected TFs and their regulated genes, we retrieve the information of corresponding proteins from the Human Protein Atlas (HPA) (https://www.proteinatlas.org/) [25], [26]. To simplify network visualization, we included only TF-regulated genes classified as unfavorable prognostic factors in the respective cancer types, as indicated in the pathology section of the HPA. The visualization of the gene regulatory networks was constructed by Cytoscape (v3.10.1). In addition, immunohistochemistry (IHC) staining images for the selected transcription factors were obtained from the HPA. The procedures for IHC image generation and staining intensity evaluation were described in their previous publication [26]. To ensure fair comparison, we selected images generated using the same antibody for each target gene.

### 2.12 Similar compound recognition using the LINCS L1000 database

To investigate the compounds that have similar functions to *P. chinensis*, a comparative analysis between the gene expression profiles of A549 cells in response to *P. chinensis* and different compounds was conducted. The comparative analysis was performed using the GSEA algorithm based on the perturbation-driven gene expression of A549 cells in LINCS L1000 gene expression resources (level 5 data) (http://clue.io/) [27]. This resource encompasses extensive gene expression profiles resulting from compound perturbations in cultured human cells and level 5 data provides the normalized z-score of different gene expression values compared to the control group.

Briefly, the top 500 up-regulated and the top 500 down-regulated genes of each compound were selected as predefined gene sets based on the value of z-score. The DEGs of the *P. chinensis* treatment were sorted in descending order based on the log_2_ fold change. Then, GSEA was applied to analyze the enrichment score of each compound-related gene set based on the ranked gene list of DEGs. Here, a positive normalized enrichment score (NES) indicates that the gene set was activated and vice versa. The summarized NES for each compound was determined by subtracting the NES of the top 500 down-regulated genes from the NES of the top 500 up-regulated genes. Finally, the compound's similarity with *P. chinensis* was ranked by the summarized NES value. The p-values corresponding to the NES scores of the top 500 up-regulated and top 500 down-regulated genes were corrected by the Benjamini-Hochberg procedure.

### 2.13 Pathway activities assessment using PROGENy

We employed PROGENy analysis to infer the activities of 14 cancer-related pathways induced by herbal treatments [28]. The top 100 genes of each pathway were selected as signatures. The R package progeny (v2.0.1) was used to calculate the z-score of the pathway’s activities based on the test statistic (the stat column of DESeq2 results).

### 2.14 Molecular docking

The molecular docking calculations were based on crystallographic data for the protein structure of MDM2 binding to the transactivation domain of p53 (PDB:1YCR), they were optimized by the Protein Preparation Wizard module at pH 7.0. Then, prepared ligands were docked to the optimized protein by Glide with Standard Precision mode. All other parameters for the above process were the default parameters. The docking studies were performed by Maestro of Schrödinger Suites (version 2020–4), and obtained poses were analyzed with PyMOL (version 2.3.0).

### 2.15 Reporter metabolites

Reporter metabolites analysis was performed using the reporter Metabolites function in the RAVEN Toolbox version2.0, based on the Human-GEM model (version 1.15)[29]. Differentially expressed genes were mapped to the metabolic network, and reporter metabolites were identified as those surrounded by significant transcriptional changes.

### 2.16 Statistical analysis

DEG analysis was done by the R package DESeq2 (v1.36.0). Statistical differences in cell viability between herbal treatment groups and the control group were estimated by *t*-test. Error bars shown in the graphs are presented as the mean ± standard deviation. P-values less than 0.05 are considered statistically significant and shown by an asterisk mark (∗).

## 3 Results

### 3.1 *P. chinensis*, *B. chinense,* and *P. umbellatus* showed superior anticancer effects on A549 and Huh7 cells among the 14 herbs tested

**Fig. 1:**
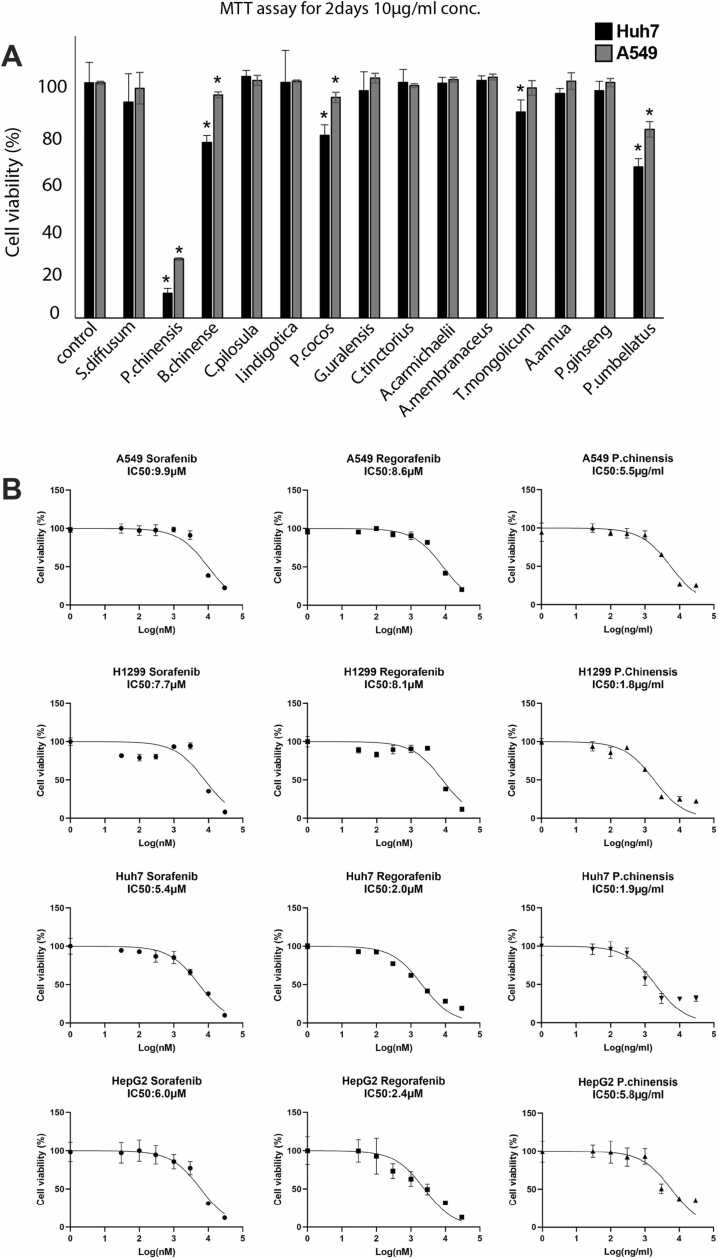
*P. chinensis*, *B. chinense,* and *P. umbellatus* showed superior anticancer effects on A549 and Huh7 cells among the 14 herbs tested.(A)Cell viability of 10 µg/ml herbal extracts treated on A549 and Huh7 cells for 48 h. (B)IC_50_ of *P. chinensis* on A549, H1299, Huh7 and HepG2 cells. Sorafenib and Regorafenib were used as positive controls.

We treated both A549 and Huh7 cells with 14 different herbal extracts, including *H. diffusa, P.chinensis, B. chinense, S. miltiorrhiza, C. cyrtophyllum, Poria, G. uralensis, C. tinctorius, A. carmichaelii, A. membranaceus, Taraxacum, A. carvifolia, P. ginseng,* and *P. umbellatus.* Based on cell viability assays, three herbs—*P. chinensis*, *B. chinense*, and *P. umbellatus*— demonstrated the strongest inhibitory effects on both A549 and Huh7 cell viability (Fig. 1A). Among them, *P. chinensis* showed the most potent suppression in A549 cells and Huh7 cells. To further evaluate its efficacy, we determined the IC50 value of *P. chinensis*, using two multi-target kinase inhibitors—Sorafenib and Regorafenib—as positive controls(Fig. 1B). Since A549 Is a p53 wild-type cell line and Huh7 harbors a p53 mutation, we also included H1299 (p53-null) for lung cancer and HepG2 (p53 wild-type) for liver cancer to investigate whether the treatment effect of *P. chinensis* extends to cancer cell lines with different p53 mutation statuses. These results suggest that *P. chinensis* exhibits a strong inhibitory effect on cancer cell viability across different cell lines.

### 3.2 Differential responses of A549 and Huh7 cells to herbal treatments

To understand the anti-cancer mechanism of *P. chinensis, B. chinense* and *P. umbellatus* in A549 and Huh7 cancer cell lines, we performed transcriptomic analysis. PCA plots showed that the gene expression profiles of different treatment groups were distinctly separated by their cell type (Supplementary Figure 1A). A heatmap was generated to visualize the expression profiles of the top 50 most variable genes across all samples, revealing distinct cell–type–specific expression patterns independent of the herbal treatments (Supplementary Figure 1B).

**Fig. 2:**
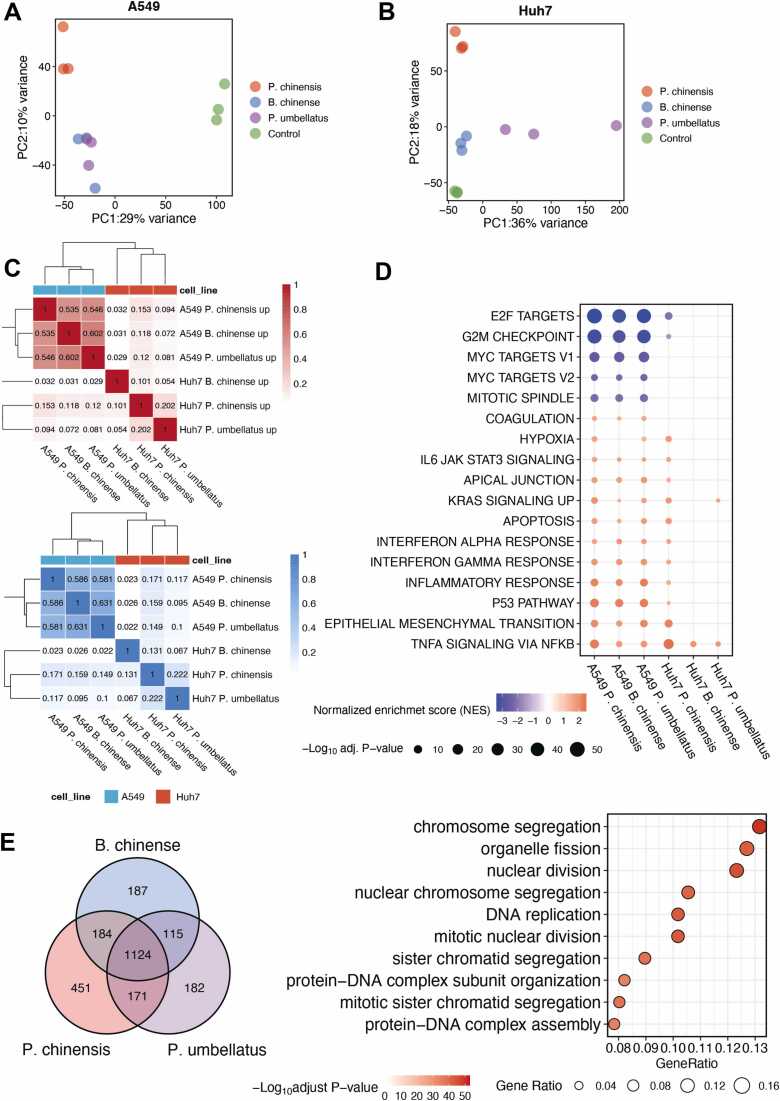
The treatment effect of *P. chinensis*, *B. chinense*, and *P. umbellatus* on A549 cells exhibits a high degree of similarity(A)Principal Component Analysis (PCA) plot displays RNA-seq gene expression in A549 cells treated with *P. chinensis*, *B. chinense*, *P. umbellatus*, and a control group.(B)Principal Component Analysis (PCA) plot displays RNA-seq gene expression in Huh7 cells treated with *P. chinensis*, *B. chinense*, *P. umbellatus*, and a control group.(C)Heatmaps of the Jaccard index shows the similarity of up and down-regulated genes across different herbal treatment groups in both A549 and Huh7 cells(D)A dot plot illustrates the results of Gene Set Enrichment Analysis (GSEA) on different herbal treatment groups in both A549 and Huh7 cells according to the MSigDB hallmark gene set. The dot size reflects the adjusted p-value, and the dot colour indicates the normalized enrichment score (red represents up-regulated pathways and blue represents down-regulated pathways)(E)The common genes that are downregulated following treatment with *P. chinensis*, *B. chinense*, and *P. umbellatus* in A549 cells. The Gene Set representative analysis (GSOA) is conducted based on the biological process category from the Gene Ontology (GO) dataset. The size of the dot indicates the gene ratio (the DEGs assigned to the corresponding pathway relative to the total analysed DEGs), and the colour of the dot indicates the adjusted p-value.

In both A549 and Huh7 cells, PCA analysis revealed that samples treated with *P. chinensis*, *B. chinense*, and *P. umbellatus* clustered separately from the control groups, indicating significant alterations in gene expression following herbal treatment. the *P. chinensis* group displayed distinct PC2 values in both A549 and Huh7 cells compared to *B. chinense* and *P. umbellatus*, suggesting that the transcriptional response induced by *P. chinensis* may differ from those elicited by the other two herbs. These findings are consistent with the cell viability results, which showed that *P. chinensis* exerted the strongest cytotoxic effect among the three herbs. We observed distinct gene expression changes under the treatment of *P. chinensis, B. chinense,* and *P. umbellatus* compared to control group, based on an adjusted p-value cutoff of 0.05. These gene expression changes are detailed in the volcano plots (Supplementary Figure 2). We applied the Jaccard index to assess the similarity of DEGs across different treatment groups, which also revealed cell type–specific differences. The three herbs induced a greater number of overlapping genes in A549 cells compared to Huh7 cells (Fig. 2C).

Next, we applied GSEA to investigate the functional pathways enriched among the DEGs in each treatment group. The results revealed that the herbal treatments induced distinct pathway alterations between the two cell lines (Fig. 2D). In A549 cells, cell cycle arrest–related pathways—including the G2/M checkpoint, E2F targets, and MYC targets—were significantly downregulated following treatment with the three herbs. In contrast, the p53 signaling pathway and apoptosis, both essential for inhibiting DNA replication and cell division, were upregulated. In contrast, Huh7 cells displayed a divergent response, with enrichment of cell cycle and division-related pathways observed only following *P. chinensis* treatment. We also applied GSOA focusing on the biological process category, which yielded similar results (Supplementary Figures 3–5). Given the substantial overlap in DEGs following the three herbal treatments in A549 cells, we performed enrichment analysis on the shared DEGs to better understand the common molecular effects of these treatments. As shown in Supplementary Figure 6 and Fig. 2E, a total of 980 genes were commonly upregulated and 1124 genes were commonly downregulated in A549 cells following the three herbal treatments. Notably, the shared downregulated DEGs were significantly enriched in pathways such as organelle fission, nuclear division, chromosome segregation, and DNA replication—all of which are associated with cell division.

### 3.3 *P. chinensis* treatment downregulates cell cycle–related transcription factors in A549 and Huh7 Cells

**Fig. 3:**
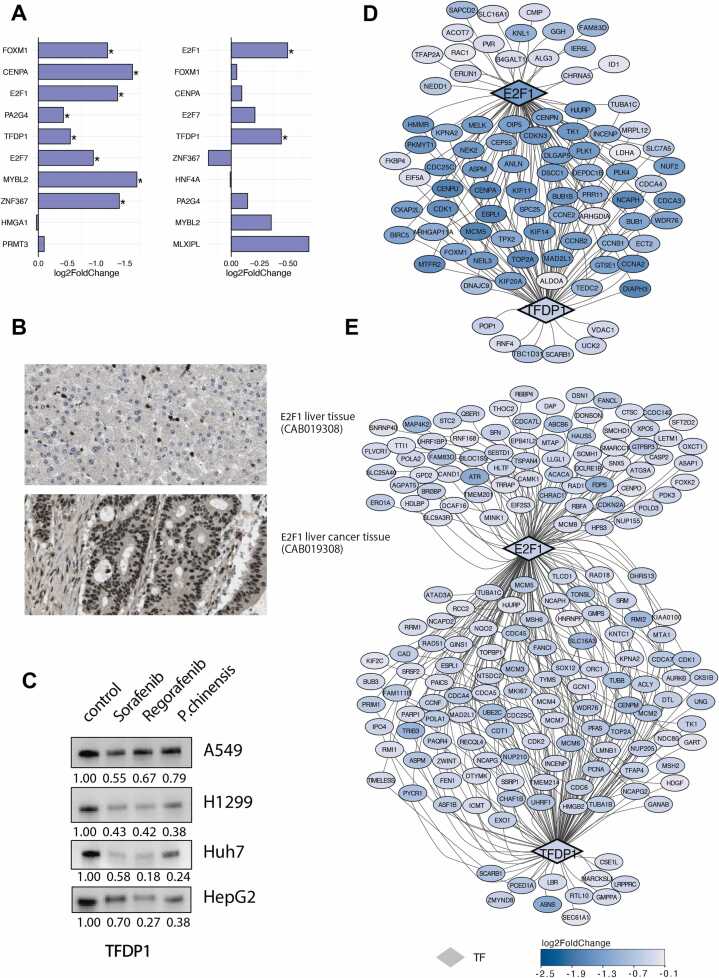
*P. chinensis* induced similar TFs changes in A549 and Huh7 cells(A)ChEA3 enrichment reveals the top ten TFs enriched by down-regulated genes after treatment with *P. chinensis* in A549 (left) and Huh7 cells (right), respectively. TFs are ranked by average integrated ranks across libraries. TFs labelled with an asterisk (*) exhibit significant downregulation following *P. chinensis* treatment in comparison to the control group(B)IHC staining of E2F1 protein in liver and liver cancer tissue(antibody:CAB019308). All figures have been sourced from the Human Protein Atlas (HPA).(C)Western blot analysis of TFDP1 protein expression in A549, H1299, Huh7, and HepG2 cells following treatment with control, sorafenib, regorafenib, and P. chinensis. (D-E) The regulatory pathways are governed by E2F1 and TFDP, along with their downstream regulated genes in A549 (D) and Huh7 cells (E), respectively. The intensity of the colour assigned to each gene in the visualization corresponds to the log2 fold change values from the differential expression analysis. The colour blue is used to denote genes that have been downregulated.

The above results showed that *P. chinensis* presented the most toxic effect among the three herbs. Next, we investigated the TFs changes associated with *P. chinensis* treatment in both A549 and Huh7 cells. ChEA3 was applied to identify the TFs responsible for regulating the up- and down-regulated DEGs. The identified top ten TFs in A549 and Huh7 cells were detailed in Supplementary Table 1. Notably, eight of the top ten TFs related to the down-regulated genes in A549 and Huh7 cells induced by *P. chinensis* treatment were the same (Fig. 3A). This observation is noteworthy considering the relatively low similarity observed in the down-regulated genes between these two cell lines following *P. chinensis* treatment. We also found relatively low similarities between genes regulated by these eight TFs between A549 and Huh7 cells. The Jaccard index of genes regulated by the same TFs in A549 and Huh7 cells ranged from 0.106 to 0.362 (Supplementary Figure 7C).

**Fig. 4:**
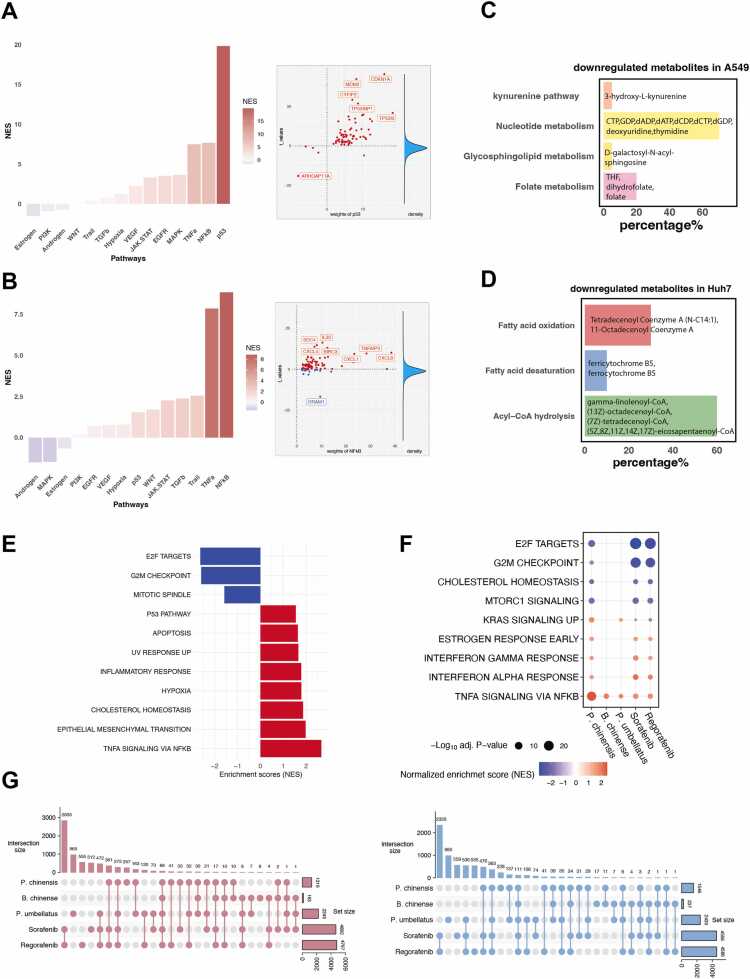
*P. chinensis* exhibited anti-cancer effects through the MDM2-p53 pathway in A549 cell and TNF-α-induced NF-κB in Huh7 cell(A–B) Bar plots showing the normalized enrichment scores (NES) from PROGENy analysis in A549 and Huh7 cells, illustrating the activation of 14 key tumor-associated pathways following *P. chinensis* treatment. Dot plots visualizing the most responsive genes in the p53 pathway (A549) and NF-κB pathway (Huh7) after P. chinensis treatment.(C-D) Top 20 downregulated metabolites following P. chinensis treatment in A549 and Huh7 cells, respectively.(E) Bar plot showing the results of Gene Set Enrichment Analysis (GSEA) based on DEGs from the comparison of P. chinensis with B. chinense and P. umbellatus in A549 cells. Bar length represents the normalized enrichment score (NES), and dot color indicates pathway direction—red for upregulated and blue for downregulated pathways. GSEA was performed using the MSigDB Hallmark gene sets.(F) Dot plot illustrating the results of GSEA for different herbal and chemotherapy treatment groups in Huh7 cells compared to the control group, based on the MSigDB Hallmark gene set.(G) UpSet plots show the intersection size of up- and downregulated genes among *P. chinensis*, *B. chinense*, *P. umbellatus*, *Sorafenib,* and *Regorafenib* treatment groups in Huh7 cells.

Among the eight common transcription factors, most were associated with the regulation of the cell cycle. Notably, E2F1 and TFDP1 were significantly downregulated in both A549 and Huh7 cells. We further retrieved the protein information of these TFs from HPA. Survival analysis indicated that two of these TFs are associated with poor prognosis in cancer. Additionally, IHC staining from the HPA showed higher protein expression levels of these TFs in tumor tissues compared to normal tissues (Fig. 3B). TFDP1 forms heterodimers with E2F transcription factors, enhancing their DNA-binding capacity and transcriptional activation of target genes involved in cell cycle progression. We further validated the changes in TFDP1 expression by Western Blot analysis. As shown in Fig. 3C, *P. chinensis* treatment led to a decrease in TFDP1 expression in both A549 and Huh7 cells, suggesting impaired formation of functional E2F/DP complexes. We also examined TFDP1 expression in H1299 and HepG2 cells, which also showed reduced expression following *P. chinensis* treatment. Then, We constructed transcription factor regulatory networks for A549 and Huh7 cells, focusing on the two identified TFs and their downstream target genes. As shown in Figs. 4D and 4E, the regulatory networks differed notably between the two cell lines, both in the composition and the number of genes regulated by these TFs.

### 3.4 *P. chinensis* exhibited anti-cancer effects through the p53 signaling pathway in A549 cells and through TNF-α/NF-κB signaling in Huh7 cells

We applied enrichment analyses, including GSOA and GSEA, to explore pathway-level expression changes following *P. chinensis* treatment (Fig. 2). The results revealed distinct enrichment patterns between A549 and Huh7 cells. To further investigate the activity of cancer-related signaling pathways, we performed PROGENy analysis, which provided additional insights into the pathway-specific effects of *P. chinensis*. The results showed that the p53 pathway was the most prominently activated among all pathways in A549 cells (Fig. 4A). We visualized the p53 most responsive genes, which showed the key genes, such as *CDKN1A*, *TP53I3*, and *TP53INP1*, contributing most to the pathway enrichment. To investigate why *P. chinensis* presented a higher toxic effect than the other two herbs in A549 cells, we conducted a DEGs analysis comparing *P. chinensis* with *B. chinense* and *P. umbellatus*. GSEA was performed using these DEGs to identify the pathways specifically enriched following *P. chinensis* treatment. The results showed that, compared to the other two herbs, *P. chinensis* more strongly downregulated E2F targets and the G2/M checkpoint, while upregulating apoptosis-related pathways in A5499 cells. Notably, activation of the p53 pathway was also more pronounced in the *P. chinensis* group (Fig. 4E). We also performed reporter metabolite analysis to investigate key metabolic changes following *P. chinensis* treatment in A549 cells. The results showed that *P. chinensis* predominantly downregulated metabolites involved in nucleotide metabolism (e.g., CTP, dGDP, dTDP), folate metabolism (e.g., THF, folate), and the kynurenine pathway (3-hydroxy-L-kynurenine) (Fig. 4C). Notably, these metabolite changes align well with the known regulatory functions of the p53 pathway. Previous studies have reported that p53 can suppress nucleotide [30] and folate metabolism [31] by downregulating key metabolic enzymes, thereby limiting DNA synthesis and promoting cell cycle arrest. Furthermore, p53 has also been shown to modulate the kynurenine pathway [32], which plays a crucial role in immune regulation.

In Huh7 cells, PROGENy analysis showed that *P. chinensis* mostly affected the activity of TNF-α and NF-κB pathway (Fig. 4B). To better understand the treatment effect of *P. chinensis*, we conducted transcriptomic analysis on Huh7 cells treated with *Sorafenib* and *Regorafenib*, which are multi-target kinase inhibitors that are commonly employed in liver cancer therapy. Upset plots showed the number of DEG intersections between three herbs, *Sorafenib* and *Regorafenib* (Fig. 4G). GSEA results showed that *P. chinensis* had a stronger effect in regulating TNF-α signalling via NF-κB compared to *B. chinense*, *P. umbellatus, Sorafenib* and *Regorafenib*. (Fig. 4F). Metabolite analysis revealed that fatty acid oxidation–related metabolic pathways were downregulated, potentially limiting the energy supply available to cancer cells [33]. Additionally, several acyl-CoA intermediates were markedly reduced. This decrease in acyl-CoA levels may impair fatty acid oxidation and disrupt membrane-associated signaling, thereby further inhibiting cancer cell proliferation [34], [35].

### 3.5 A compound similarity analysis using the LINCS L1000 database identified *P. chinensis* as having a treatment effect profile similar to that of MDM2–p53 interaction inhibitors

To further explore the molecular mechanisms underlying the effects of *P. chinensis* treatment, we searched for compounds that exhibit similar transcriptional responses. Specifically, we conducted a comparative analysis between the gene expression profiles induced by *P. chinensis* and those of various small-molecule compounds in A549 cells, using the LINCS L1000 database. Due to limited compound-induced gene expression data available for Huh7 cells, only A549 data were used in this analysis. Similarity between *P. chinensis* and each compound was evaluated using GSEA, as detailed in the Methods section. After filtering 53,827 compounds, we identified the top ten compound signatures with the highest similarity scores to *P. chinensis* (Table 2). These ten signatures were associated with six unique compounds, including AMG232, clofarabine, nutlin-3, CX-5461, mitoxantrone, and mericitabine, of which five are anticancer agents. Notably, AMG232 appeared twice, nutlin-3 appeared three times within the top ten signatures, and both AMG232 and nutlin-3 were inhibitors of the p53-MDM2 protein-protein interaction.

**Table 2:** Top-ten compound perturbations similar to *P. chinensis* treatment in A549 cells based CMap database.

Rank	Batch	CMap name	Dose	Treat time	Cell	NES (top 500 genes)	p-value of NES (top 500 genes)	NES (bottom 500 genes)	p-value of NES (bottom 500 genes)	Total NES
**1**	b40	AMG−232	10 μM	24 h	A549	2.349660	8.05e−26	−3.710580	1.84e−130	6.060239
**2**	b41	clofarabine	10 μM	48 h	A549	2.491568	2.03e−35	−3.545747	2.21e−113	6.037314
**3**	b34	AMG−232	10 μM	24 h	A549	2.325613	2.26e−26	−3.688789	5.59e−125	6.014401
**4**	b42	nutlin−3	10 μM	24 h	A549	2.242212	9.75e−24	−3.736071	5.39e−140	5.978283
**5**	b32	CX−5461	10 μM	24 h	A549	2.337019	1.17e−28	−3.622920	3.44e−118	5.959940
**6**	b32	mitoxantrone	1.11 μM	24 h	A549	2.258089	4.12e−23	−3.697765	1.91e−124	5.955854
**7**	b40	nutlin−3	10 μM	24 h	A549	2.244571	2.78e−24	−3.698417	9.92e−131	5.942989
**8**	b32	mitoxantrone	0.37 μM	24 h	A549	2.320273	3.24e−27	−3.617512	2.80e−125	5.937785
**9**	b41	nutlin−3	10 μM	48 h	A549	2.187997	2.33e−22	−3.717155	5.28e−135	5.905152
**10**	b41	mericitabine	10 μM	48 h	A549	2.314613	4.39e−26	−3.581254	9.96e−112	5.895867

**Fig. 5:**
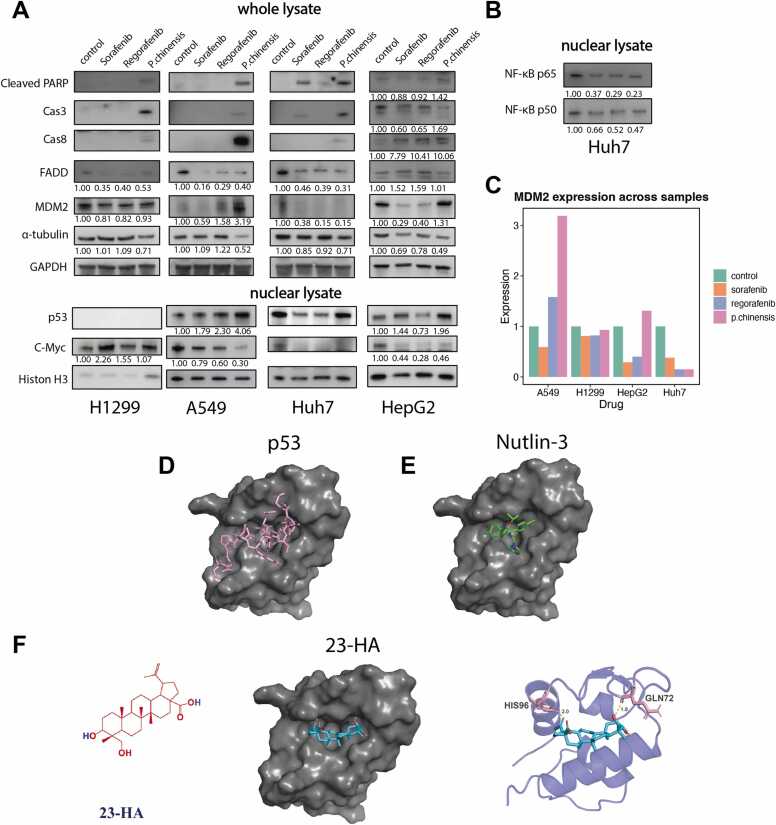
*P. chinensis* as having a treatment effect similar to MDM2-p53 interaction inhibitors(A) Western blot analysis of apoptosis- and p53-related proteins in H1299, A549, Huh7, and HepG2 cells.(B) Western blot analysis of NF-κB–related proteins in Huh7 cells.(C) Bar plot illustrating MDM2 expression levels across different groups. (D) Interaction between p53 (pink) and MDM2 (grey surface representation), illustrating the native binding conformation.(E) Docking result of Nutlin-3 (green), a known p53-MDM2 inhibitor, showing its ability to occupy the key binding site and disrupt the interaction.(F) The structures of 23-hydroxybetulinic acid (23-HA). The docking result of 23-HA demonstrates its binding within the p53-MDM2 pocket, suggesting potential inhibitory activity.

To further validate the finding of our computational analysis, we investigated the expression levels of apoptotic proteins using western blot (Fig. 5A). We found that *P. chinensis* induced strong apoptotic protein expression, including cleaved PARP, active caspase-3, and active caspase-8 both in A549 and Huh7 cells, compared to both *Sorafenib* and *Regorafenib*. What’s more, our similar compound recognition algorithm showed that the therapeutic function of *P. chinensis* was similar to MDM2-p53 binding inhibitors. Previous studies have shown that disruption of this interaction leads to an increase in p53, then activating the p53-mediated apoptotic response. [36] Additionally, in a negative feedback loop, p53 transcriptionally upregulates MDM2 levels [37]. In our study, nuclear protein Western Blot analysis revealed that *P. chinensis* treatment in A549 cells led to a 4.06-fold increase in p53 levels and a marked reduction in the oncogenic downstream effector c-Myc (to 0.3-fold of the original level). Notably, MDM2 protein expression was markedly elevated in A549 cells following *P. chinensis* treatment (Fig. 5C). In addition, protein expression levels were examined in H1299 and HepG2 cells to assess the influence of p53 mutation status. *P. chinensis* treatment upregulated MDM2 expression in A549 and HepG2 cells, both of which are p53 wild-type, but not in H1299 and Huh7 cells, which lack functional p53. These findings suggest that *P. chinensis* exerts its effects through the p53 pathway primarily in p53 wild-type cells, with a more pronounced impact observed in lung cancer cells.

*P. chinensis* is rich in Triterpenoid saponins (TSs), such as anemoside B4 (AB4), anemoside A3 (AA3), and 23-hydroxybetulinic acid (23-HA). These three molecules share a similar steroid core. Therefore, we selected 23-HA as a representative compound for molecular docking analysis due to its structural similarity to the other two molecules. Fig. 5D illustrates the interaction between p53 (pink) and the MDM2 protein (grey). Nutlin-3, a well-known inhibitor of the p53-MDM2 interaction, exhibits a similar effect to *P. chinensis* as predicted by our algorithm. As shown in Fig. 5E, Nutlin-3 effectively occupies the key binding cavity of the p53-MDM2 complex. Similarly, our docking analysis (Fig. 5F) indicates that 23-HA can also bind to this pocket, suggesting its potential inhibitory effect on the p53-MDM2 interaction.

## 4 Discussion

In this study, three herbs *P. chinensis*, *B. chinense*, and *P. umbellatus* demonstrated promising anticancer effects. We conducted a comprehensive transcriptomic analysis to investigate the molecular mechanisms underlying the efficiency of these herbs. Notably, among the three herbs studied, *P. chinensis* showed the highest efficacy in treating both lung and liver cancer. We performed a comprehensive transcriptomic analysis to investigate the underlying mechanisms of herbal treatment efficacy. Additionally, we developed a similar compound recognition approach using the LINCS L1000 database to infer the treatment targets of *P. chinensis.* This database operates by mapping "query signatures" to reference expression profiles within the dataset, allowing for the evaluation of their similarity and the identification of potential drugs that may influence specific cellular states [38]. The identified targets were subsequently validated through experimental assays and molecular docking analyses.

In A549 cells, we observed activation of the p53 pathway following *P. chinensis* treatment. Furthermore, we identified two anticancer agents, AMG232 and Nutlin-3, which exhibited gene expression profiles similar to those induced by *P. chinensis*. Notably, both compounds are designed to inhibit the p53-MDM2 protein-protein interaction. Western blot analysis confirmed the activation of the p53-MDM2 pathway exclusively in A549 cells, consistent with our computational findings. Molecular docking analyses further revealed that the active compound of *P. chinensis* could bind to the same pocket on the MDM2 protein as Nutlin-3, effectively inhibiting the interaction between p53 and MDM2. We further validated this finding by including two additional cell lines with different p53 mutation statuses, which confirmed that P. chinensis exerts its anticancer effects primarily through the p53 pathway in p53 wild-type cells. Interestingly, the effect was more pronounced in the lung cancer cell line A549 compared to the liver cancer cell line HepG2.

The p53 protein is a transcription factor that plays a critical role in tumour suppression. It can activate various genes in response to DNA damage, leading to growth arrest or apoptosis. This action prevents damaged cells from proliferating and transmitting mutations to future generations [39]. Due to its vital role in maintaining genomic integrity, p53 is often referred to as the “guardian of the genome” [40]. The MDM2 protein acts as a negative regulator of p53. It binds to the transcriptional activation domain of p53, inhibiting its ability to regulate target genes and exert antiproliferative effects [41]. Therefore, compounds that prevent the interaction between p53 and MDM2 are considered a promising strategy for activating p53-related tumour-suppression activity [39]. Several natural products have been reported to have antitumor effects targeting the p53-MDM2 pathway. For example, Isokotomolide A, extracted from the leaves of Cinnamomum kotoense was reported to inhibit cell growth and induce cell cycle arrest by decreasing the interaction of p53-MDM2 [42]. Besides, several network pharmacological analyses revealed that the efficacy of Baitouweng (*P. chinensis*) decoction in treating radiation enteritis and ulcerative colitis was linked to its interaction with p53 targets [43] or the enhancement of the p53 signalling pathway [44].

In Huh7 cells, we found another pathway affected by *P. chinensis* treatment, which was the TNF-α induced NF-κB pathway. TNFα is an inflammatory cytokine that often serves as the activator of NF-κB [45]. NF-κB could induce anti-apoptotic genes and then enhance cancer cell proliferation [46]. In our study, treatment with *P. chinensis* resulted in decreased NF-κB expression levels and increased apoptotic proteins. Because of the absence of sufficient compound perturbation data on Huh7 cells in the LINCS L1000 database, we didn’t perform analysis to identify the compounds with similar targets as *P. chinensis* on Huh7 cells. Nonetheless, *P. chinensis* demonstrated a promising potential for cancer treatments, but relevant targets and mechanisms still need to be explored.

Disruption of the normal regulation of cell cycle progression and division is a critical event that drives the transformation of normal cells into malignant ones [47]. Our findings demonstrate that *P. chinensis* exerts anticancer effects by downregulating cell cycle–related transcription factors, including E2F1 and TFDP1, across all examined cell lines. Interestingly, the downstream mechanisms triggered by the *P. chinensis* treatments displayed distinct profiles between A549 and Huh7 cells. There is extensive crosstalk between the E2F1 and p53, both of which influence critical cellular decisions. They share common upstream regulators, and E2F1 can indirectly affect the levels and activity of p53 by upregulating proteins that stabilize and activate it [48]. A close relationship between E2F1 and NF-κB has also been reported. Dysregulated E2F1 can promote apoptosis and enhance cellular sensitivity to pro-apoptotic stimuli by disrupting NF-κB signaling [49]. The precise underlying mechanisms remain to be fully elucidated.

Our study has several limitations. First, although the herbs demonstrated anticancer efficacy in vitro, further in vivo and preclinical studies are necessary to evaluate their safety, effectiveness, and potential interactions with conventional cancer therapies. Second, we used crude herbal extracts containing complex mixtures of phytochemicals, which makes it challenging to identify the specific active compounds. Future studies should aim to isolate and investigate individual components for more precise mechanistic insights. Third, while we inferred metabolic changes based on transcriptomic data, direct metabolomic analyses are needed to validate these findings. Finally, our findings highlighted the important role of the E2F1/TFDP1 complex in the response to *P. chinensis* treatment. However, further studies, such as investigating promoter occupancy, are needed to better elucidate the underlying regulatory mechanisms.

## 5 Conclusion

In our study, we identified *P. chinensis* as a noteworthy herbal candidate for the treatment of lung and liver cancer. Our integrative approach, combining computational analysis and experimental validation, has revealed that *P. chinensis* exhibits anti-cancer properties by inducing apoptosis through the p53 and TNF-α/NF-κB pathways in A549 and Huh7 cells, respectively. Future clinical research is essential to deepen our understanding of the relevant biological mechanisms.

## CRediT authorship contribution statement

**Xiya Song:** Writing – review & editing. **Xiangtai Kong:** Writing – review & editing, Methodology. **Yuefeng Bi:** Writing – review & editing. **Hasan Turkez:** Writing – review & editing. **Ling Fu:** Writing – review & editing. **Chengxue Pan:** Writing – review & editing. **Mathias Uhlen:** Writing – review & editing. **Mengzhen Li:** Writing – original draft, Software, Methodology, Formal analysis, Conceptualization. **Hongmin Liu:** Writing – review & editing. **Cheng Zhang:** Writing – review & editing, Supervision. **Hong Yang:** Writing – review & editing, Methodology. **Adil Mardinoglu:** Writing – review & editing, Supervision. **Han Jin:** Writing – review & editing, Methodology. **Woonghee Kim:** Writing – original draft, Methodology.

## Author statement

All authors have read and approved the submission of this manuscript to the Computational and Structural Biotechnology Journal. AM is the co-founder of SZA Longevity Inc. and Trustlife Global Inc. The other authors declare that they have no competing interests.

## Ethics approval and consent to participate

Not applicable

## Consent for publication

Not applicable

## Funding

This work was financially supported by 10.13039/501100004063Knut and Alice Wallenberg Foundation (No: 72110). Mengzhen Li is being sponsored in her doctoral study by the 10.13039/501100004543China Scholarship Council (Grant No. 202208440189).

## Declaration of Competing Interest

The authors declare the following financial interests/personal relationships which may be considered as potential competing interests: AM is the co-founder of SZA Longevity Inc. and Trustlife Global Inc. The other authors declare that they have no competing interests.

## Data Availability

The RNA-seq data generated for this study have been deposited in the Gene Expression Omnibus database under accession code GSE250410.
