# Supplementary material for: Anticancer effects and mechanisms of Pulsatilla chinensis, Bupleurum chinense and Polyporus umbellatus on human lung carcinoma and hepatoma cells
Source: Comput Struct Biotechnol J. 2025 Jul 13;27:3066–78. doi: 10.1016/j.csbj.2025.07.023 (PMC12281591; doi:10.1016/j.csbj.2025.07.023)
Supplement: Supplementary file 1 — Supplementary material [file mmc1.docx]

**Supplementary Table 1. Top10 enriched TFs predicted by ChEA3 after *P. chinensis* treatment**

| **enriched by down-regulated genes** | **A549** | | | **Huh7** | |
| --- | --- | --- | --- | --- | --- |
|  | **Rank** | **TF** | **Description** | **TF** | **Description** |
|  | 1 | FOXM1 | Forkhead box M1 | E2F1 | E2F transcription factor 1 |
|  | 2 | CENPA | Centromere protein A | FOXM1 | Forkhead box M1 |
|  | 3 | E2F1 | E2F transcription factor 1 | CENPA | Centromere protein A |
|  | 4 | PA2G4 | Proliferation-associated 2G4 | E2F7 | E2F transcription factor 7 |
|  | 5 | TFDP1 | Transcription factor Dp-1 | TFDP1 | Transcription factor Dp-1 |
|  | 6 | E2F7 | E2F transcription factor 7 | ZNF367 | Zinc finger protein 367 |
|  | 7 | MYBL2 | MYB proto-oncogene like 2 | HNF4A | hepatocyte nuclear factor 4 A |
|  | 8 | ZNF367 | Zinc finger protein 367 | PA2G4 | Proliferation-associated 2G4 |
|  | 9 | HMGA1 | High mobility group AT-hook 1 | MYBL2 | MYB proto-oncogene like 2 |
|  | 10 | PRMT3 | Protein arginine methyltransferase 3 | MLXIPL | MLX-interacting protein-like |
| **enriched by up-regulated genes** | **Rank** | **TF** | **Description** | **TF** | **Description** |
|  | 1 | SMAD3 | SMAD Family Member 3 | CSRNP1 | Cysteine And Serine Rich Nuclear Protein 1 |
|  | 2 | ELK3 | ETS Transcription Factor ELK3 | JUN | Jun Proto-Oncogene, AP-1 Transcription Factor Subunit |
|  | 3 | FOSL2 | FOS Like 2, AP-1 Transcription Factor Subunit | FOSL1 | FOS Like 1, AP-1 Transcription Factor Subunit |
|  | 4 | FOXD1 | Forkhead Box D1 | FOSB | FosB Proto-Oncogene, AP-1 Transcription Factor Subunit |
|  | 5 | ATF3 | Activating Transcription Factor 3 | FOSL2 | FOS Like 2, AP-1 Transcription Factor Subunit |
|  | 6 | NFKB2 | Nuclear Factor Kappa B Subunit 2 | KLF6 | KLF Transcription Factor 6 |
|  | 7 | GLMP | Glycosylated Lysosomal Membrane Protein | ATF3 | Activating Transcription Factor 3 |
|  | 8 | SNAI2 | Snail Family Transcriptional Repressor 2 | NR4A3 | Nuclear Receptor Subfamily 4 Group A Member 3 |
|  | 9 | FOSL1 | FOS Like 1, AP-1 Transcription Factor Subunit | SNAI1 | Snail Family Transcriptional Repressor 1 |
|  | 10 | HIF1A | Hypoxia Inducible Factor 1 Subunit Alpha | JUNB | JunB Proto-Oncogene, AP-1 Transcription Factor Subunit |

**Supplementary Table 2. Top10 enriched TFs predicted by ChEA3 after *B. chinense*** **treatment**

| **enriched by down-regulated genes** | **A549** | | | **Huh7** | |
| --- | --- | --- | --- | --- | --- |
|  | **Rank** | **TF** | **Description** | **TF** | **Description** |
|  | 1 | FOXM1 | Forkhead box M1 | CREB3L3 | CAMP Responsive Element Binding Protein 3 Like 3 Pr |
|  | 2 | CENPA | Centromere protein A | ATF5 | Activating Transcription Factor 5 |
|  | 3 | E2F1 | E2F transcription factor 1 | MLXIPL | MLX Interacting Protein Like |
|  | 4 | PA2G4 | Proliferation-associated 2G4 | ARID3C | AT-Rich Interaction Domain 3C |
|  | 5 | E2F7 | E2F transcription factor 7 | FOXA3 | Forkhead Box A3 |
|  | 6 | TFDP1 | Transcription Factor Dp-1 | NR1H4 | Nuclear Receptor Subfamily 1 Group H Member 4 |
|  | 7 | MYBL2 | MYB proto-oncogene like 2 | HNF4A | Hepatocyte Nuclear Factor 4 Alpha |
|  | 8 | ZNF367 | Zinc finger protein 367 | NR1I2 | Nuclear Receptor Subfamily 1 Group I Member 2 |
|  | 9 | PRMT3 | Protein Arginine Methyltransferase 3 | ETV4 | ETS Variant Transcription Factor 4 |
|  | 10 | HMGA1 | High Mobility Group AT-Hook 1 | ZBED3 | Zinc Finger BED-Type Containing 3 |
| **enriched by up-regulated genes** | **Rank** | **TF** | **Description** | **TF** | **Description** |
|  | 1 | SMAD3 | SMAD Family Member 3 | FOSB | FosB Proto-Oncogene, AP-1 Transcription Factor Subunit |
|  | 2 | GLMP | Glycosylated Lysosomal Membrane Protein | CSRNP1 | Cysteine And Serine Rich Nuclear Protein 1 |
|  | 3 | GLIS2 | GLIS Family Zinc Finger 2 | ATF3 | Activating Transcription Factor 3 |
|  | 4 | TEAD1 | TEA Domain Transcription Factor 1 | FOS | Fos Proto-Oncogene, AP-1 Transcription Factor Subunit |
|  | 5 | SNAI2 | Snail Family Transcriptional Repressor 2 | NR4A3 | Nuclear Receptor Subfamily 4 Group A Member 3 |
|  | 6 | ELK3 | ETS Transcription Factor ELK3 | JUN | Jun Proto-Oncogene, AP-1 Transcription Factor Subunit |
|  | 7 | FOXD1 | Forkhead Box D1 | ZBTB21 | Zinc Finger And BTB Domain Containing 21 |
|  | 8 | CREB3L2 | CAMP Responsive Element Binding Protein 3 Like 2 | EPAS1 | Endothelial PAS Domain Protein 1 |
|  | 9 | NFKB2 | Nuclear Factor Kappa B Subunit 2 | BHLHE40 | Basic Helix-Loop-Helix Family Member E40 |
|  | 10 | RARG | Retinoic Acid Receptor Gamma | ETS2 | ETS Proto-Oncogene 2, Transcription Factor |

**Supplementary Table 3. Top10 enriched TFs predicted by ChEA3 after *P. umbellatus* treatment**

| **enriched by down-regulated genes** | **A549** | | | **Huh7** | |
| --- | --- | --- | --- | --- | --- |
|  | **Rank** | **TF** | **Description** | **TF** | **Description** |
|  | 1 | FOXM1 | Forkhead box M1 | CENPX | Centromere protein X |
|  | 2 | CENPA | Centromere protein A | THAP7 | THAP domain containing 7 |
|  | 3 | E2F1 | E2F transcription factor 1 | ZBTB45 | Zinc finger and BTB domain containing 45 |
|  | 4 | PA2G4 | Proliferation-associated 2G4 | ZNF787 | Zinc finger protein 787 |
|  | 5 | E2F7 | E2F transcription factor 7 | ZNF444 | Zinc finger protein 444 |
|  | 6 | TFDP1 | Transcription Factor Dp-1 | ZNF511 | Zinc finger protein 511 |
|  | 7 | MYBL2 | MYB proto-oncogene like 2 | MAZ | MYC associated zinc finger protein |
|  | 8 | ZNF367 | Zinc finger protein 367 | ZNF408 | Zinc finger protein 408 |
|  | 9 | HMGA1 | High Mobility Group AT-Hook 1 | SLC2A4RG | SLC2A4 regulator |
|  | 10 | ZNF695 | Zinc finger protein 695 | ZNF580 | Zinc finger protein 580 |
| **enriched by up-regulated genes** | **Rank** | **TF** | **Description** | **TF** | **Description** |
|  | 1 | FOXD1 | Forkhead box D1 | ZBED6 | Zinc finger BED-type containing 6 |
|  | 2 | ZNF469 | Zinc finger protein 469 | ZNF654 | Zinc finger protein 654 |
|  | 3 | SMAD3 | SMAD family member 3 | RBAK | RB associated KRAB zinc finger |
|  | 4 | ELK3 | ETS transcription factor ELK3 | ZNF148 | Zinc finger protein 148 |
|  | 5 | ZNF697 | Zinc finger protein 697 | ZNF12 | Zinc finger protein 12 |
|  | 6 | SNAI2 | Snail family transcriptional repressor 2 | ZNF507 | Zinc finger protein 507 |
|  | 7 | CREB3L2 | CAMP responsive element binding protein 3 like 2 | EEA1 | Early endosome antigen 1 |
|  | 8 | TWIST2 | Twist family bHLH transcription factor 2 | ZBTB6 | Zinc finger and BTB domain containing 6 |
|  | 9 | GLMP | Glycosylated lysosomal membrane protein | MYSM1 | Myb like, SWIRM and MPN domains 1 |
|  | 10 | TEAD1 | TEA domain transcription factor 1 | ZBTB11 | Zinc finger and BTB domain containing 11 |

**
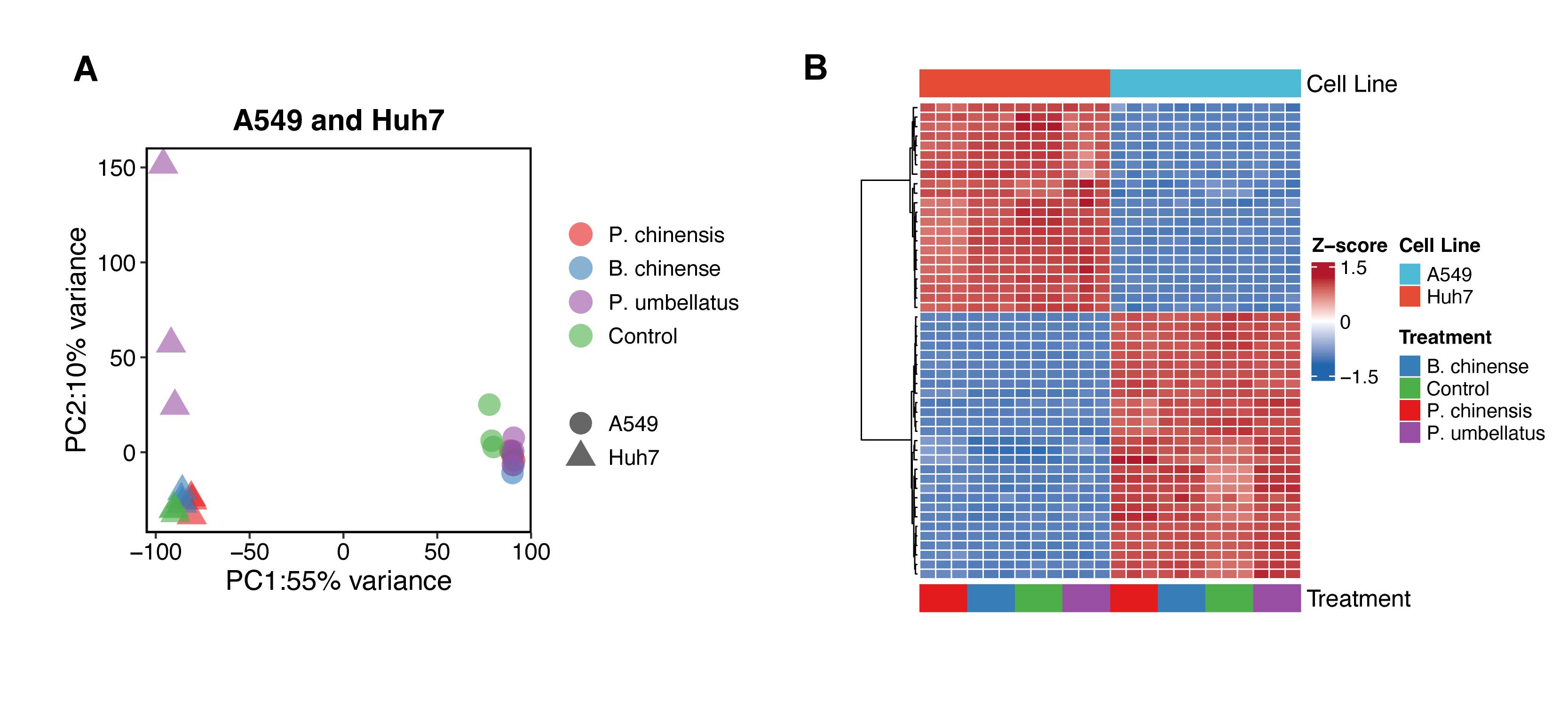
**

**Supplementary Figure 1: A549 and Huh7 cells exhibit cell type-specific expression profiles after herbal treatments**

(A) PCA plot of the RNA-seq gene expression for samples treated with *P. chinensis, B. chinense* and *P. umbellatus* and a control group in A549 and Huh7 cells. The Dot shapes denote the cell types, with circles representing A549 cells and triangles representing Huh7 cells. (B) Heatmap showing the Z-score of the top 50 genes exhibiting the highest variance in their expression levels.

**Supplementary Figure 2: Volcano plots of differential expression genes (DEGs) induced by *P. chinensis*, *B. chinense*, and *P. umbellatus* treatment in A549 and Huh7 cells**

Volcano plots showing up-(red) and downregulated (blue) genes when comparing *P. chinensis* versus control, *B. chinense* versus Control and *P. umbellatus* versus Control in A549 cells and Huh7 cells, respectively (p-value < 0.05 is used for calculating significance). In A540 cells, we observed that there were 1764, 1491, and 1518 genes up-regulated and 1930, 1610, and 1592 genes down-regulated, respectively. Similarly, in Huh7 cells, the treatments resulted in the up-regulation of 1319, 163, and 2245 genes and the down-regulation of 1546, 237, and 2430 genes, respectively.


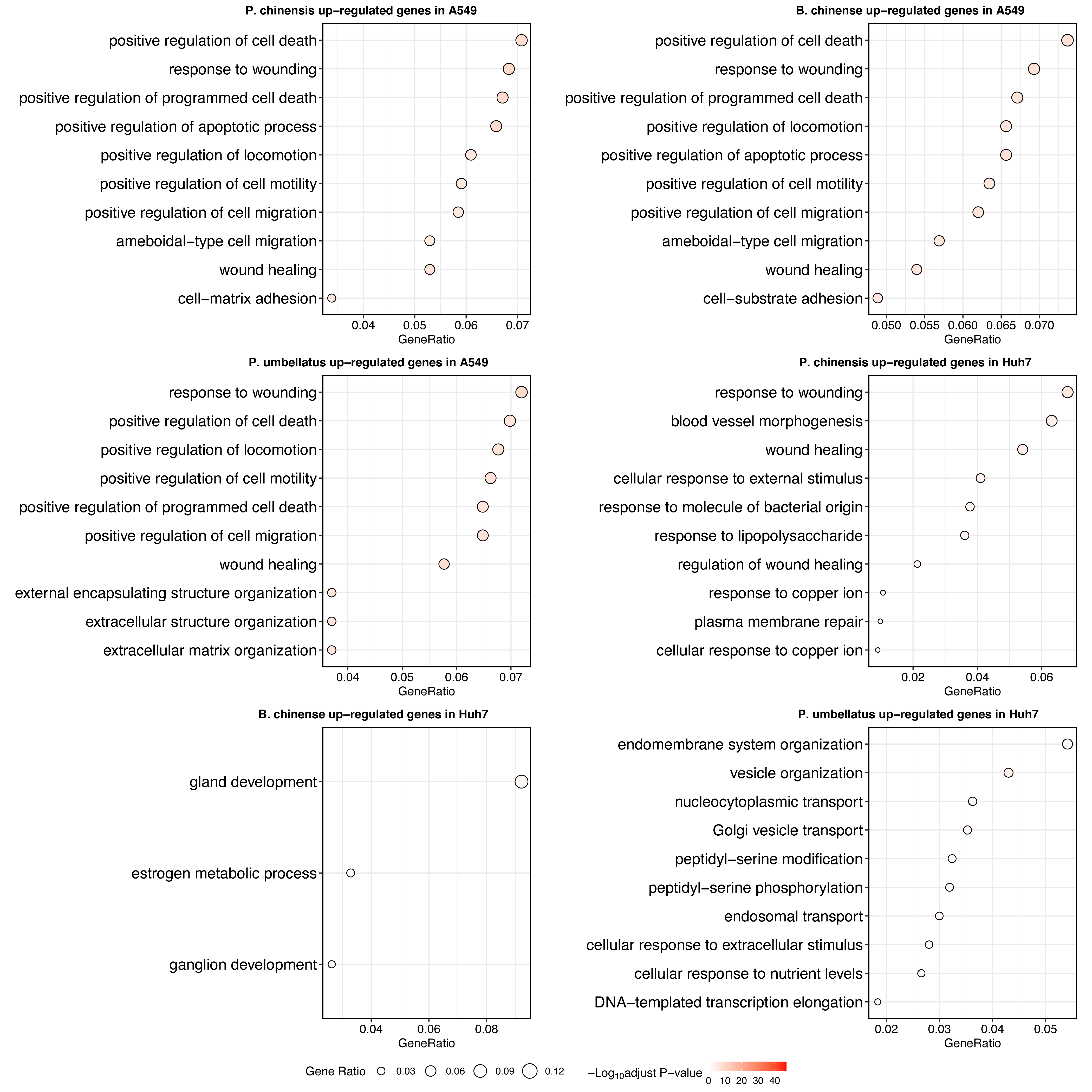


**Supplementary Figure 3: Gene Set representative analysis (GSOA) showing the up-regulated pathway after *P. chinensis*, *B. chinense*, and *P. umbellatus* treatment in A549 and Huh7 cells**

The GSOA was conducted based on the biological process category form Gene Ontology (GO) dataset. The size of the dot indicates the gene ratio, i.e., the DEGs assigned to the corresponding pathway relative to the total analysed DEGs, and the dot's colour indicates the adjusted p value.


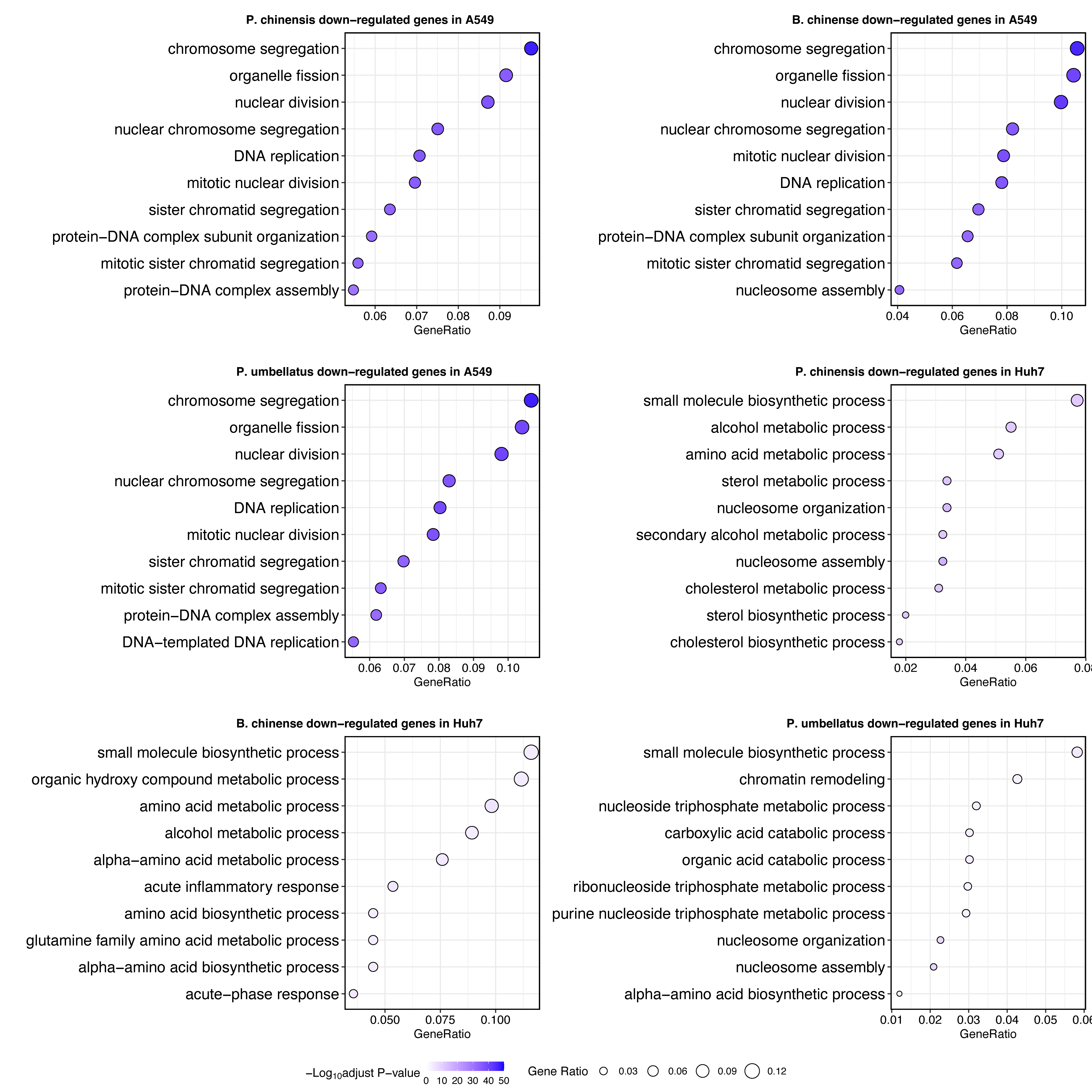


**Supplementary Figure 4: Gene Set representative analysis (GSOA) showing the downregulated pathway after *P. chinensis*, *B. chinense*, and *P. umbellatus* treatment in A549 and Huh7 cells**

The GSOA was conducted based on the biological process category from the Gene Ontology (GO) dataset. The size of the dot indicates the gene ratio, i.e., the DEGs assigned to the corresponding pathway relative to the total analysed DEGs, and the dot's colour indicates the adjusted p-value.

**Supplementary Figure 5: Heatmaps of the GO semantic similarity scores**

These plots show the concordance of enriched GO pathways across different herbal treatment groups, especially in A549 cells.


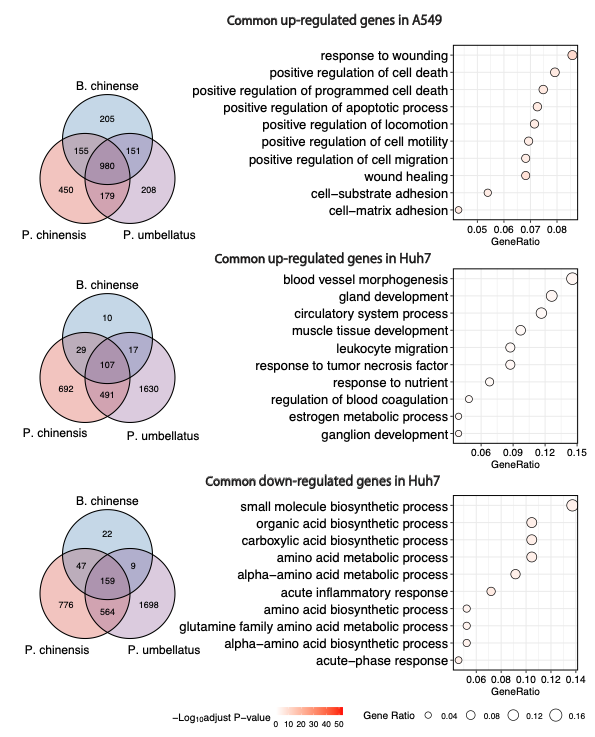


**Supplementary Figure 6:** **The common** **DEGs induced by *P. chinensis*, *B. chinense*, and *P. umbellatus* treatment and relevant enriched pathways**

Figures illustrate the common genes that are either upregulated or downregulated following treatment with *P. chinensis*, *B. chinense*, and *P. umbellatus* in A549 and Huh7 cells respectively.

The Gene Set representative analysis (GSOA) is conducted based on the biological process category from the Gene Ontology (GO) dataset. The size of the dot indicates the gene ratio (the DEGs assigned to the corresponding pathway relative to the total analysed DEGs) and the colour of the dot indicates the adjusted p-value.

The Gene Set representative analysis (GSOA) is conducted based on the biological process category from the Gene Ontology (GO) dataset. The size of the dot indicates the gene ratio (the DEGs assigned to the corresponding pathway relative to the total analysed DEGs) and the colour of the dot indicates the adjusted p-value.

Specifically, common up-regulated genes in A549 cells were significantly enriched in pathways related to cell death. The enrichment results suggested that the utilization of these three herbs in lung cancer could potentially promote cancer cell death and inhibit cancer cell division.

**Supplementary Figure 7: immunohistochemistry (IHC) staining of E2F1 and TFDP1 protein expression in normal and tumor tissue from lungs and livers.**

Heatmap of the Jaccard index shows the similarity of TFs-regulated genes between A549 and Huh7 cells
